# *EGFR* gene amplification is related to adverse clinical outcomes in cervical squamous cell carcinoma, making the EGFR pathway a novel therapeutic target

**DOI:** 10.1038/bjc.2011.222

**Published:** 2011-07-05

**Authors:** K Iida, K Nakayama, M T Rahman, M Rahman, M Ishikawa, A Katagiri, S Yeasmin, Y Otsuki, H Kobayashi, S Nakayama, K Miyazaki

**Affiliations:** 1 Department of Obstetrics and Gynecology, Shimane University School of Medicine, Enyacho 89-1, Izumo, Shimane 6938501, Japan; 2 Department of Pathology, Seirei Hamamatsu General Hospital, Hamamatsu 4308558, Japan; 3 Department of Obstetrics and Gynecology, Seirei Hamamatsu General Hospital, Hamamatsu 4308558, Japan

**Keywords:** cervical cancer, EGFR, gene amplification, survival, squamous cell carcinoma, adenocarcinoma/adenosquamous carcinoma

## Abstract

**Background:**

The aim of this study was to investigate the patterns of epidermal growth factor receptor (EGFR) overexpression, *EGFR* gene amplification, and the presence of activating mutations in the tyrosine kinase domain of this gene in squamous cell carcinomas and adenocarcinomas/adenosquamous carcinomas of the uterine cervix.

**Methods:**

The EGFR expression, amplification, and mutation in cervical carcinomas were assessed by immunohistochemistry, fluorescence *in situ* hybridisation, and PCR–SSCP, respectively, and correlated with clinical data collected by a retrospective chart review. A functional assessment was performed by inactivating EGFR in cervical cancer cells with the potent inhibitor AG1478.

**Results:**

Immunohistochemical analysis revealed that 6 out of 59 (10.2%) cervical squamous cell carcinomas showed significant amplification of the *EGFR* locus, whereas none of the 52 adeno/adenosquamous cell carcinomas had detectable *EGFR* amplification (*P*<0.05). The *EGFR* amplification significantly correlated with shorter overall survival (*P*=0.001) in cervical squamous cell carcinomas. Multivariate analysis showed that *EGFR* gene amplification was an independent prognostic factor for overall survival (*P*=0.011). None of the squamous cell carcinomas (0%: 0 out of 32) had detectable oncogenic mutations in *EGFR* exons 18 through 21. The frequencies of *KRAS* and *BRAF* mutations were very low in both squamous and adeno/adenosquamous cell carcinomas. Sensitivity of cervical cancer cells to AG1478 depended on the presence of EGFR overexpression. AG1478-induced EGFR inactivation in cell lines with EGFR overexpression significantly suppressed tumour development and progression in a mouse xenograft model.

**Conclusion:**

Our data suggest that EGFR signalling is important in a subset of cervical squamous cell carcinomas and that anti-EGFR therapy may benefit patients who carry the 7p11.2 amplicon in their tumours.

Uterine cervical cancer is the second most common malignancy among women worldwide (Jones, 1999). Despite the availability of screening, cervical cancer is still a leading cause of cancer death in Japanese women. This is partly because some patients continue to present with advanced-stage disease for which conventional therapy is less effective. Therefore, novel therapeutic agents are urgently needed to improve the outcome in these patients.

Human papillomavirus (HPV) is the aetiologic agent of cervical cancer; however, HPV infection is not sufficient. Alterations in oncogenes and tumour-suppressor genes in cervical cells are essential for cervical carcinogenesis. Amplification of DNA in certain chromosomal regions is one of the mechanisms by which genes that are critical in the development and progression of human cancers are activated (Schwab, 1999). Numerous oncogenes and other cancer-related genes have been identified in these amplified regions. In squamous cervical cancers in particular, proto-oncogenes, such as *EGFR* (7q12), *MYC* (8q24), *ERBB2* (17q11.2-12), *CCND1* (11q13), *HRAS* (11q15.5), and *cIAP1* (11q22) are often activated by amplification (Ocadiz *et al*, 1987; Pfeiffer *et al*, 1989; Hale *et al*, 1993; Mitra *et al*, 1994; Kurzrock *et al*, 1995; Imoto *et al*, 2002). Some of these genes are clearly associated with malignant phenotypes (Pfeiffer *et al*, 1989; Hale *et al*, 1993; Imoto *et al*, 2002). These oncogenes, however, are only a subset of the genes present in the amplified regions. To gain new insight into the molecular pathogenesis of cervical cancer, and to establish diagnostic markers and therapeutic targets, more genes within these amplified regions must be characterised.

The gene for the epidermal growth factor receptor (EGFR) maps to 7p11.2-p12 and comprises 28 exons (Hynes and Lane, 2005), which encode a protein containing an extracellular ligand-binding domain, a transmembrane domain, and a tyrosine kinase domain (Hynes and Lane, 2005). Epidermal growth factor receptor was the first tyrosine kinase transmembrane receptor to be directly linked with human cancer (Hynes and Lane, 2005). In recent years, EGFR tyrosine kinase inhibitors have received FDA (Food and Drug Administration) approval and are currently being tested in patients with lung, gastric, and breast cancer (Baselga and Arteaga, 2005). There appear to be distinct mechanisms for EGFR activation in different types of human neoplasms. Gene amplification of *EGFR* has been described in oligodendrogliomas (Fallon *et al*, 2004), glioblastomas (Marquez *et al*, 2004), lung carcinomas (Baselga and Arteaga, 2005; Giaccone, 2005), gastric carcinomas (Takehana *et al*, 2003), and recently, in breast carcinomas (Al-Kuraya *et al*, 2004). The *EGFR*-activating mutations are present in a subset of central nervous system tumours and lung cancer (Giaccone, 2005; Hynes and Lane, 2005), but are remarkably rare in breast cancer cell lines and human breast cancer samples (Bhargava *et al*, 2005). Previous studies have shown EGFR to be frequently overexpressed in primary cervical cancer (Pfeiffer *et al*, 1989; Hale *et al*, 1993; Kristensen *et al*, 1996; Scambia *et al*, 1998; Kersemaekers *et al*, 1999; Ngan *et al*, 2001; Gaffney *et al*, 2003); however, the mechanism of EGFR activation (i.e., gene amplification or activating mutation) in cervical cancer is poorly understood. Additionally, the EGFR/RAS/RAF/MEK/ERK pathway and its downstream effectors have primarily been studied in the context of squamous cell carcinomas, which comprise 85–90% of cervical cancers (Pappa *et al*, 2006). It is unclear whether EGFR amplification is a feature of adenocarcinomas and adenosquamous carcinomas, which comprise 10–25% of cervical cancers (Smith *et al*, 2000; Chan *et al*, 2003; Arias-Pulido *et al*, 2008).

The aim of this study was to investigate the differences in EGFR overexpression, *EGFR* gene amplification, and activating mutations in the tyrosine kinase (TK) domain of this gene between squamous cell carcinomas and adenocarcinomas/adenosquamous carcinomas of the uterine cervix. In addition, we compared the phenotypes in cultured cervical cancer cells with various EGFR expression levels after treatment with the potent EGFR inhibitor AG1478.

## Materials and methods

### Tissue samples

A total of 59 paraffin-embedded tumour tissue samples were obtained from the Department of Obstetrics and Gynecology at Shimane University Hospital; all samples were cervical squamous cell carcinomas. Also, 52 adenocarcinomas/adenosquamous carcinomas were obtained from the Department of Obstetrics and Gynecology at Seirei Hamamatsu General Hospital. Patients had received appropriate therapy at either Shimane University Hospital or Seirei Hamamatsu General Hospital between January 1994 and December 2007. Tumour staging was performed according to the International Federation of Gynecology and Obstetrics (FIGO) classification (Shepherd, 1996). The invasive squamous cell carcinomas consisted of 26 cases of stage I disease, 11 of stage II disease, 17 of stage III disease, and 5 of stage IV disease. All tumours were classified histologically according to the World Health Organization criteria. The median patient age was 60 years (range 26–84 years). The invasive adenocarcinomas/adenosquamous cell carcinomas consisted of 38 cases of stage I disease, 8 of stage II disease, 5 of stage III disease, and 1 of stage IV disease. All tumours were classified histologically according to the World Health Organization criteria. The median patient age was 46 years (range 27–82 years).

Stage I and II patients were treated with class II or class III radical hysterectomies with pelvic lymph node dissection. Stage I patients with positive lymph node metastasis or positive lymphovascular space invasion and all stage II patients received concurrent chemoradiotherapy or radiotherapy as adjuvant therapy. Stage III and IV patients were treated with concurrent chemoradiotherapy or radiotherapy alone.

Patients with an incomplete response to radiotherapy and patients with recurrent tumours were treated with a variety of salvage chemotherapy agents, including cisplatin, peplomycin, and paclitaxel. The follow-up period ranged from 5 to 120 months, with a median of 45 months. Acquisition of tissue specimens and clinical information was approved by an institutional review board (Shimane University and Seirei Hamamatsu General Hospital). Only patients with follow-up data were included. The paraffin tissue blocks were organised into tissue microarrays, each made by removing 3 mm diameter cores of tumour from the block. Selection of the area to core was made by a gynaecologic oncologist (KN) and pathology technician (KI) and was based on a review of the H&E slides.

### Fluorescence *in situ* hybridisation

The BAC clones (RP11-81B20 and CTD-2199A14) containing the genomic sequences of the 7p11.2 amplicon were purchased from Bacpac Resources (Children's Hospital, Oakland, CA, USA) and Invitrogen (Carlsbad, CA, USA). The Bac clones corresponding to Ch7q11.2 (RP11-91E1) were used to generate reference probes. RP11-91E1 was labelled by nick translation with biotin-dUTP; RP11-81B20 and CTD-2199A14 were labelled similarly with digoxigenin-dUTP. To detect biotin-labelled and digoxigenin-labelled signals, slides were first incubated with FITC-avidin (Vector Laboratories, Burlingame, CA, USA) and a digoxigenin-coupled mouse antibody (Roche Molecular Biochemicals, Mannheim, Germany). Slides were subsequently incubated with a biotinylated avidin antibody (Vector Laboratories) and tetramethylrhodamine B isothiocyanate (TRITC)-conjugated rabbit anti-mouse antibody (Sigma, St Louis, MO, USA). The final incubation was with FITC-avidin and TRITC-conjugated goat anti-rabbit antibody (Sigma). Slides were counterstained with 4′,6′-diamidino-2-phenylindole (Sigma).

Fluorescence *in situ* hybridisation (FISH) signals were evaluated with an Olympus fluorescence microscope BX41 (Tokyo, Japan) by two individuals who were blind to the treatment history of each patient. Separate narrow band-pass filters were used for detection of tetramethylrhodamine B isothiocyanate, FITC, and 4′,6′-diamidino-2-phenylindole signals. Using × 60 objective lens, ∼100 tumour cells were examined for each specimen, and the numbers of fluorescent signals within tumour cells from the *EGFR* gene BAC probe and chromosome 7q11.2 reference BAC probe were recorded. Amplification of *EGFR* was defined as a ratio of *EGFR* BAC probe signals to chromosome 7q11.2 reference BAC probe signals of 2 : 1 or more.

### Immunohistochemistry

Immunohistochemistry was performed on deparaffinised sections after treatment with a 0.4% pepsin/0.01 N HCl (Sigma-Aldrich, Diessenhofen, Germany) solution using an EGFR antibody (clone 31G7; Zymed, Invitrogen, Carlsbad, CA, USA) at a dilution of 1 : 10, followed by incubation with a biotinylated linker and streptavidin-horseradish peroxidase (LSAB2 system-HRP, DAKO Cytomation, Carpinteria, CA, USA). The signals were visualised using ABC^+^ (DAKO Cytomation) as the substrate–chromagen at room temperature for 10 min. Slides for all samples were evaluated with a light microscope by two researchers; the researchers were blind to the clinicopathological factors. Immunoreactivity was scored by two investigators as follows: 0, undetectable; 1+, weakly positive; 2+, moderately positive; and 3+, intensely positive. In normal cervical epithelium, EGFR immunoreactivity was not detectable (immunointensity score=0).

### DNA isolation

Serial 10 *μ*m unstained sections of paraffin blocks were cut, and one adjacent haematoxylin and eosin-stained section was taken for identification and selection of the tumour tissue. Areas where tumour cells represented at least 85% of the total area were marked, and using a sterile needle, gross macroscopic dissection was performed. The dissected tissues were placed in microcentrifuge tubes, and DNA isolation was performed as described previously (Nakayama *et al*, 2001b).

### Screening for EGFR mutations

Screening for *EGFR* (exons 18–21) mutations was done by PCR–single-strand conformational polymorphism (PCR–SSCP). The PCR conditions were as follows: After denaturation at 95 °C for 5 min, DNA amplification was performed in 35 cycles consisting of denaturation at 95 °C for 30 s, primer annealing at 58 °C for 1 min, and extension for 1 min at 72 °C. Nested PCR was used for EGFR exons 18–21. The PCR primers for the EGFR mutational analysis are listed in Supplementary Table 1. The PCR products (20 *μ*l) were incubated at 95 °C for 10 min with an equal volume of formamide loading buffer (98% formamide, 10 mM EDTA, and 1 mg ml^–1^ bromophenol blue and xylene cyanol). The SSCP gels were run at 20 °C. Samples with an SSCP pattern different from the normal pattern were directly sequenced. The SKOV3 DNA, which was wild type for EGFR exons 18–21 (Lynch *et al*, 2004), served as the normal control. All cases were confirmed twice with a new PCR amplification, SSCP, and direct sequencing analysis. All positive results were confirmed twice with an independent PCR amplification, followed by direct sequencing.

### Mutational analysis of *KRAS* and *BRAF*

Polymerase chain reaction was then performed followed by nucleotide sequencing using the iCycler (Bio-Rad, Hercules, CA, USA). Exon 1 of *KRAS* and exon 15 of *BRAF* were both sequenced, as these mutational hot spots, together, harbour nearly all published mutations (Nakayama *et al*, 2006). The primers for PCR and sequencing were manufactured by GeneLink (Hawthorne, NY, USA), and their sequences are described in a previously published report (Nakayama *et al*, 2006). The sequences were analysed using the Lasergene program, DNASTAR (Madison, WI, USA).

### Cell culture and cell lines

Human cervical adenocarcinoma cell lines (Hela, Hela TG, and Hela P3) were obtained from Tohoku University (Sendai, Japan). Human cervical squamous cell carcinoma cell lines ME180 and CaSki were also obtained from Tohoku University, whereas SKGIIIa, SKGIIIb, HCS2, and BOKU (also squamous) were obtained from the Health Science Research Resources Bank (Tokyo, Japan). All human cervical cancer cell lines were maintained in DMEM (Life Technologies, Gaithersburg, MD, USA) supplemented with 5% fetal bovine serum, 100 U ml^–1^ penicillin, and 100 *μ*g ml^–1^ streptomycin at 37 °C in an atmosphere of 5% CO_2_.

### Western blot analysis

Cell lysates were prepared by dissolving cell pellets in Laemmli sample buffer (Bio-Rad) supplemented with 5% *β*-mercaptoethanol (Sigma). Western blot analysis was performed on all cervical cancer cell lines. Similar amounts of total protein from each lysate were loaded and separated on 10% Tris-Glycine-SDS polyacrylamide gels (Novex, San Diego, CA, USA) and electroblotted to Millipore Immobilon-P polyvinylidene difluoride membranes (Millipore, Bedford, MA, USA). Membranes were probed with an EGFR antibody (1 : 100) (Zymed) followed by a peroxidase-conjugated anti-mouse or anti-rabbit immunoglobulin (1 : 20 000). The same membrane was probed with an antibody that reacted with GAPDH (1 : 10 000; Cell Signaling Technology, Beverly, MA, USA) for loading controls. Western blots were developed by chemiluminescence (Pierce, Rockford, IL, USA).

### Cell proliferation assay

Cells were seeded in 96-well plates at a density of 3000 cells per well and treated with or without the EGFR inhibitor AG1478 (Merck Biosciences, Darmstadt, Germany). Cell number was determined indirectly by an MTT assay (Nakayama *et al*, 2001a). Each cell line was treated with 10 *μ*mol l^–1^ AG1478 to inhibit EGFR function, and cell viability was measured using an MTT assay 98 h later. An equal amount of DMSO was used as a control. Data were expressed as the mean±1 s.d. of triplicate determinations.

### Tumour xenograft in nude mice

To confirm the *in vitro* effects of AG1478 *in vivo*, we injected 3 × 10^6^ ME180 or Hela cells into the subcutaneous tissue of *nu/nu* mice (4 weeks of age). BALB/c *nu/nu* mice, 4 weeks old, were purchased from Charles River Japan, Inc. (Kanagawa, Japan). To evaluate the efficacy of anti-EGFR therapy, mice in the tumour group were treated with intraperitoneal injections of AG1478 (10 *μ*g/treatment/week for 5 weeks) beginning 2 weeks after cell injection. Mice were killed at 12 weeks, and tumours were excised and weighed. Differences in tumour progression were tested between AG1478-treated and untreated mice using Student's *t-*test. Animal experiments were performed in accordance with the regulations of the institutional ethical commission (Shimane University) and of the United Kingdom Coordinating Committee on Cancer Research guidelines (UKCCCR, 1998).

### Statistical methods for clinical correlation

Overall survival was calculated from the date of diagnosis to the date of last follow-up. Age and performance status distributions were similar between patients expressing EGFR and those not expressing it. The data were plotted as Kaplan–Meier curves, and the statistical significance was determined by the log-rank test. Data were censored when patients were lost to follow-up. A multivariate prognostic analysis was performed using the Cox proportional hazards model. The *χ*^2^-test or Fischer's exact test was used for comparisons of categorical data. Student's test (for comparison of two groups) or one-way analysis of variance (ANOVA; for comparison of more than two groups) was used to evaluate numeric data.

## Results

### Relationship between EGFR protein expression and histological variants

**Figure 1:**
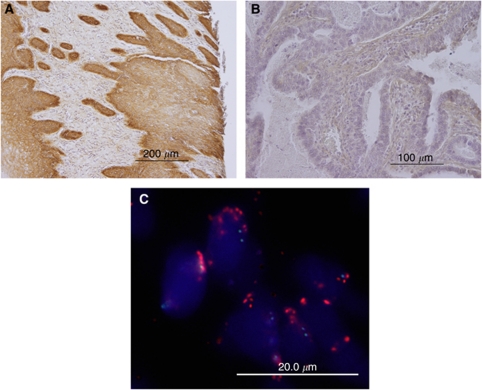
(**A**) Immunoreactivity of EGFR in cervical cancer tissues. Intense immunoreactivity is present in the cytoplasm of cervical squamous carcinoma cells (upper left panel). (**B**) A cervical adenocarcinoma case with negative staining for EGFR (upper right panel). (**C**) Dual-colour fluorescence *in situ* hybridisation (FISH) demonstrates amplification of the *EGFR* gene in cervical cancer. FISH analysis showing a homogeneously stained region in a tumour with gene amplification.

The EGFR immunoreactivity was detected in the tumour cell cytoplasm (Figure 1A). High-level EGFR expression (EGFR immunointensity of 2+ or 3+) was observed in 37% (22 out of 59) of the analysed cervical squamous cell carcinomas. High-level EGFR expression (EGFR immunointensity of 2+ or 3+) was observed in only 2.0% (1 out of 52) of the analysed cervical adenocarcinomas/adenosquamous cell carcinomas (Figure 1B). The samples fell into one of two groups depending on the status of EGFR immunostaining. High EGFR staining intensity (2+ and 3+) was more frequently found in cells of squamous cell carcinomas than in adenocarcinomas/adenosquamous cell carcinomas (*P*<0.0001, *χ*^2^-test; Table 1A).

### The frequency of EGFR gene amplification was higher in squamous cell carcinomas than in adeno/adenosquamous cell carcinomas

Out of 59 cervical squamous cell carcinomas, 6 (10.2%) showed significant amplification of *EGFR* (Figure 1C and Table 1B). None of the adenocarcinomas showed detectable *EGFR* amplification. Amplification of *EGFR* was more frequently found in cells of squamous cell carcinomas than in cells of adenocarcinomas/adenosquamous cell carcinomas (*P*<0.05, *χ*^2^-test; Table 1B).

### EGFR-activating mutations were not found in cervical carcinomas

Of the 59 cervical squamous cell carcinoma samples we examined, 32 were available for PCR–SSCP analysis. None of the squamous cell carcinomas (0%: 0 out of 32) or adeno/adenosquamous cell carcinomas (0%: 0 out of 48) had detectable oncogenic mutations in *EGFR* exons 18 through 21.

### Effect of EGFR amplification and protein expression on overall survival

**Figure 2:**
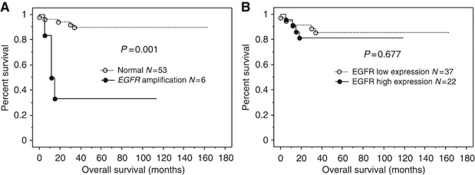
(**A**, **B**) Kaplan–Meier survival curve in 78 patients with cervical squamous cell carcinoma with respect to EGFR protein expression or gene amplification. The *EGFR gene* amplification significantly correlated with shorter overall survival (*P*=0.001). The EGFR protein expression did not correlate with shorter overall survival in patients with cervical carcinomas.

Next, we examined the prognostic value of EGFR expression levels and gene amplification. The Kaplan–Meier estimates of overall survival are plotted in Figure 2. The *EGFR gene* amplification significantly correlated with shorter overall survival (*P*=0.001). There were nonsignificant trends between high EGFR protein expression and poor overall survival (*P*=0.677). A univariate analysis demonstrated that FIGO stages III and IV (*P*=0.009, log-rank test), patient age ⩾60 years (*P*=0.041, log-rank test), and *EGFR* gene amplification (*P*=0.001, log-rank test) correlated with shorter overall survival. The multivariate analysis showed that *EGFR* gene amplification was an independent prognostic factor for overall survival (*P*=0.011; Table 2).

### The frequencies of *KRAS* and *BRAF* mutation were low in both squamous cell and adeno/adenosquamous cell carcinomas

Of the 52 cervical adenocarcinoma samples we examined, 48 were available for PCR and direct sequence analysis. Somatic mutations in *KRAS* were identified in 3 (6.3%) of 48 cervical adeno/adenosquamous cell carcinomas. In contrast, somatic mutations in *BRAF* were identified in 1 (2.1%) of 48 adeno/adenosquamous cell carcinomas. Somatic mutations in either *KRAS* or *BRAF* were identified in 4 (8.3%) of 48 adeno/adenosquamous cell carcinomas. Of the 59 cervical squamous cell carcinoma samples we examined, 32 were available for PCR direct sequence analysis. None of the squamous cell carcinomas (0%: 0 out of 32) had detectable oncogenic mutations in either *KRAS* or *BRAF.*

### Differential effects of EGFR inactivation on cervical cancer cells

**Figure 3:**
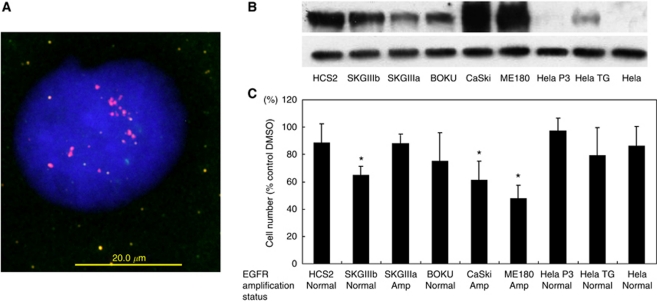
Biofunctional effects of EGFR inactivation on different cervical cancer cell lines. (**A**) Dual-colour fluorescence *in situ* hybridisation (FISH)-validated amplification of the *EGFR* gene in cervical cancer cell lines. The FISH analysis showing a homogeneously stained region in CaSki cells with gene amplification. (**B**) Western blot indicates the protein level of EGFR in each cell line. (**C**) Each cell line was treated with 10 *μ*mol l^–1^ AG1478 to inhibit EGFR function, and cell viability was measured with an MTT assay 98 h later. An equal amount of DMSO was used as a control. ^*^P<0.05 *vs* Hela P3.

It is known that oncogenic signalling caused by gene amplification or an activating mutation provides survival advantages for tumour growth. To investigate the possibility that cervical cancer cells in which the *EGFR* gene was amplified or the protein overexpressed depended on EGFR signals for survival, we analysed cervical cancer cell lines for expression levels of EGFR by western blotting (Figure 3A). Among the cell lines tested, ME180, CaSki, and SKGIIIb cells were found to carry the 7p11.2 amplicon and overexpress EGFR. HCS2 cells expressed the EGFR protein but did not contain the amplicon. Low expression levels of EGFR were detected in Hela P3, Hela TG, and Hela cells. Treatment with the EGFR inhibitor AG1478 had differential inhibitory effects on cell growth, depending on the *EGFR* status of the treated cells, with the highest drug sensitivity in ME180, CaSki, and SKGIIIb cells and the lowest in Hela P3 and Hela cells (Figure 3B).

### EGFR inactivation suggests a rational treatment for cervical cancer

**Figure 4:**
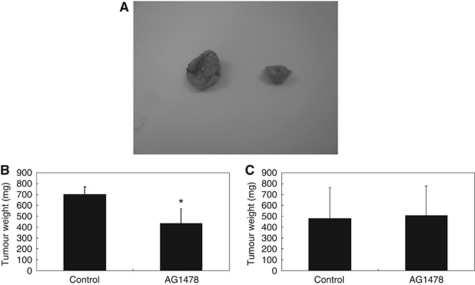
Validation of anti-EGFR therapy in a mouse xenograft cervical cancer model. Athymic nude mice were injected subcutaneously with ME180 or Hela cells. After 2 weeks, each mouse was treated with a placebo or AG1478 at a dose of 10 *μ*g/treatment/week for 5 weeks. (**A**) Appearance of the subcutaneous tumours. (**B**) The mice injected with AG1478 developed significantly smaller subcutaneous xenograft tumours than those carrying placebo-treated cells of the *EGFR-*amplified cell line ME180. (**C**) There were no differences in subcutaneous xenograft tumour weights between the AG1478-treated group and control groups transplanted with Hela cells containing wild-type, nonamplified, *EGFR*. ^*^P<0.05 *vs* control.

Based on the above findings, we investigated whether AG1478 had a growth inhibitory effect on tumour formation and development *in vivo*. Tumour xenografts from both ME180 (*EGFR* amplification) and Hela (*EGFR* wild type and without *EGFR* amplification) cell lines were established in a nu/nu mouse model. All mice carrying ME180 tumours and injected with AG1478 developed significantly smaller subcutaneous xenograft tumours than the mice treated with the placebo (Figure 4). There were no differences in subcutaneous xenograft tumour weights between the AG1478-treated group and control groups in the mice transplanted with Hela cells (Figure 4).

## Discussion

The higher frequency of EGFR expression in squamous cell carcinomas compared with adenocarcinomas/adenosquamous cell carcinomas is a finding of interest. It suggests that adenocarcinomas/adenosquamous carcinomas may be distinguished from squamous cell carcinomas based on characteristic genetic alterations. Additionally, this observation further supports the theory that cervical carcinoma arises from multiple pathways (Contag *et al*, 2004). In this model, each histological type of carcinoma develops independently and is characterised by its own molecular genetic changes and gene expression profiles (Contag *et al*, 2004).

Current treatments for advanced-stage cervical carcinoma have only limited efficacy, and the treatment of metastatic or recurrent disease is most often palliative. Targeted therapeutics represent a promising area from which to develop novel effective agents (de la Motte Rouge *et al*, 2006). Development of these targeted agents depends on the identification of molecular pathways essential for tumour growth and metastasis. A number of molecular markers have been proposed as prognostic determinants in cervical cancer, including the epidermal growth factor receptor (EGFR) family. Members of the EGFR family are involved in solid-tumour pathogenesis through cell cycle control, apoptosis, angiogenesis, and regulation of invasive and metastatic potential.

The EGFR protein is overexpressed in some cervical cancers (Pfeiffer *et al*, 1989; Hale *et al*, 1993; Kristensen *et al*, 1996; Scambia *et al*, 1998; Kersemaekers *et al*, 1999; Ngan *et al*, 2001; Gaffney *et al*, 2003). Overexpression of EGFR protein in cervical tumours has been reported to be associated with poor prognosis (Pfeiffer *et al*, 1989; Hale *et al*, 1993; Kristensen *et al*, 1996; Kersemaekers *et al*, 1999; Gaffney *et al*, 2003), although in some studies, including the present study, this association is less clear (Scambia *et al*, 1998; Ngan *et al*, 2001). Lack of a consistent association may be the result of study biases. First, differences in the methodological protocols, including the source and dilution of the antibodies and the systems used to assess positive immunohistochemical reactions, may produce significant variability among results. Second, the sample sizes of many studies, including the present one, are relatively small. Larger, prospective studies are required to definitively establish an effect of EGFR overexpression on cervical cancer prognosis.

Interestingly, we found a strong correlation between poor prognosis and *EGFR* gene amplification in patients with cervical squamous cell carcinoma. To our knowledge, this is the first report that *EGFR* gene amplification is an independent prognostic factor in cervical squamous cell carcinomas. Gene amplification is an important mechanism that allows cancer cells to increase expression of driver genes, such as oncogenes, involved in growth regulation and genes responsible for drug resistance. Therefore, detection of gene amplification in tumours may be of diagnostic, prognostic, and therapeutic relevance.

Based on the above findings, we suggest that *EGFR* amplification may play an important role in cervical squamous cell carcinoma progression and, therefore, amplification may correlate more strongly with clinical parameters than protein expression. The fact that 50 out of 59 (84%) cervical squamous cell carcinomas analysed in this study received radiotherapy may reflect *EGFR* amplification related to radiation sensitivity. As this pattern of *EGFR* gene amplification has not been previously documented with FISH in cervical carcinomas, further studies are required to confirm our results and elucidate the relationship between *EGFR* gene amplification and tumour sensitivity to radiation. Additionally, as patients with recurrent disease were also treated with various chemotherapy agents including cisplatin, pepleomycin, and paclitaxel, it is unclear if survival effects were due to *EGFR* amplification or treatment differences. To address this limitation, additional studies that analyse EGFR function in large numbers of patients treated with chemotherapeutic agents such as cisplatin and paclitaxel should be performed.

As EGFR overexpression is more prevalent than *EGFR* gene amplification, we sought to investigate whether activating mutations could constitute an alternative mechanism for EGFR overexpression as this has been reported in other solid tumours (Suzuki *et al*, 2005). We did not identify activating mutations in the tyrosine kinase domain of 32 cervical squamous cell carcinomas. Our results are in agreement with previous studies demonstrating the lack of *EGFR*-activating mutations in breast cancer (Bhargava *et al*, 2005). Furthermore, in the present study, only the *EGFR* TK domain was analysed. Although exons 18–21 are the hot spot region for *EGFR* gain-of-function mutations (Giaccone, 2005; Hynes and Lane, 2005), activating mutations in other domains of the gene cannot be excluded.

The mechanism for EGFR overexpression in the majority of cervical squamous cell carcinomas remains to be identified. It is likely that, in the majority of cases, EGFR upregulation happens at the transcriptional level (Kersting *et al*, 2004). Human papillomavirus is an aetiologic factor for cervical carcinoma, and HPV proteins seem to play an important role in EGFR expression (Bosch *et al*, 2002). The HPV E5 oncoprotein inhibits the degradation of internalised EGFR (Zhang *et al*, 2005), resulting in an increase in EGFR recycling and overexpression of EGFR. Furthermore, expression of high-risk HPV E6 has been linked to an increase in EGFR levels (Akerman *et al*, 2001), and changes in functional levels of the HPV E6/E7 proteins may alter the growth rate of cervical carcinoma cell lines by reducing the stability of EGFR at the posttranscriptional level (Hu *et al*, 1997). In light of the genomic instability induced by HPV (Duensing and Munger, 2004), it has been hypothesised that EGFR could be mutated by HPV infection. In lung cancer, 21% of patients with known HPV DNA types presented an *EGFR* TK domain mutation; no association was found between the presence of high-risk HPV DNA types and *EGFR* TK domain mutations (Shigematsu *et al*, 2005). Our results, however, demonstrate that EGFR mutation is unlikely in cervical cancer. High-risk HPV proteins may affect EGFR at both the protein and gene copy number levels without gene mutation. Although amplification was documented in 10% of the squamous carcinomas in this study, we were not able to correlate the presence of *EGFR* amplification with HPV infection. Further studies are needed to establish whether a correlation exists between *EGFR* amplification and specific HPV subtypes including 16 and 18.

Recent reports have shown that *EGFR* mutations are rare or occur at a very low frequency in acute leukaemia, glioblastoma, and colorectal, gastric, breast, and hepatocellular carcinomas. Although 80% of lung cancer patients who are carriers of *EGFR* TK domain mutations experience partial responses or marked clinical improvement with gefitinib or erlotinib, patients without such mutations are refractory to these agents (Lynch *et al*, 2004). In the present study, we demonstrated that cervical squamous cell carcinoma cell lines with *EGFR* amplification were more sensitive to a potent EGFR inhibitor AG1478, which suggests that TKI therapy may have some utility in cervical cancer tumours without mutations, provided that *EGFR* amplification is present. Cetuximab, a chimeric IgG1 monoclonal antibody, and panitumumab, a fully humanised IgG2 monoclonal antibody, belong to a new generation of drugs that block extracellular ligand binding to EGFR. Cetuximab, an FDA-approved drug, has shown promising results in colorectal and head and neck cancers (Labianca *et al*, 2007). Furthermore, cetuximab has antitumour activity in NSCLC models expressing both wild-type and mutated *EGFR* (Steiner *et al*, 2007). Cervical cancer cell lines derived from primary and recurrent tumours have also been shown to be very sensitive to cetuximab-mediated antibody-dependent cellular cytotoxicity and to cetuximab-mediated inhibition of tumour growth (Bellone *et al*, 2007). Although studies in colorectal carcinoma have shown that somatic *KRAS* mutation is associated with resistance to cetuximab (Khambata-Ford *et al*, 2007; Lievre *et al*, 2008), the present study has shown *KRAS* mutations to be rare in cervical carcinomas. On the basis of these findings, cetuximab therapy may be efficacious in cervical carcinoma patients who have EGFR protein overexpression without *KRAS* mutations, particularly those who have not responded to standard treatment modalities.

## Figures and Tables

**Table 1:** The relationship between (A) EGFR expression and (B) EGFR gene amplification and cervical carcinoma histological subtype

	**Negative**	**Positive**	***P-*value**
*(A)*			
SCC	37 (62%)	22 (38%)	<0.0001
AC/ASC	51(98%)	1(2%)	
			
	**Normal**	**Amplification**	***P-*value**
*(B)*			
SCC	53 (90%)	6 (10%)	<0.05
AC/ASC	52(100%)	0 (0%)	

Abbreviations: AC=adenocarcinoma; ASC=adenosquamous carcinoma; EGFR=epidermal growth factor receptor; SCC=squamous cell carcinoma.

**Table 2:** Univariate and multivariate analyses of prognostic factors in patients with cervical squamous cell carcinoma

**Factors**	**Patients**	**Univariate hazard ratio**	**95% CI**	***P-*value**	**Multivariate hazard ratio**	**95% CI**	***P-*value**
*Patient age*							
>60 years old	29	8.7	1.1–69.2	0.041	3.9	0.3–48.1	0.287
<60 years old	30						
							
*FIGO stage*							
I, II	37	8.1	1.7–39.0	0.009	2.7	0.4–20.0	0.329
III, IV	22						
							
*EGFR FISH*							
Amplification	6	10	2.6–38.4	0.001	6.4	1.5–27.0	0.011
Normal	53						

Abbreviations: CI=confidence interval; EGFR=epidermal growth factor receptor; FIGO=International Federation of Gynecology and Obstetrics; FISH=fluorescence *in situ* hybridisation.
